# Trends in Active Surveillance for Men With Intermediate-Risk Prostate Cancer

**DOI:** 10.1001/jamanetworkopen.2024.29760

**Published:** 2024-08-22

**Authors:** Marshall A. Diven, Lhaden Tshering, Xiaoyue Ma, Jim C. Hu, Christopher Barbieri, Timothy McClure, Himanshu Nagar

**Affiliations:** 1 New York Presbyterian-Brooklyn Methodist Hospital, Brooklyn, New York; 2 New York Presbyterian Weill Cornell Medical Center, New York, New York; 3 Department of Population Health Sciences, Division of Biostatistics, Weill Cornell Medicine, New York, New York; 4 Department of Urology, Weill Cornell Medicine, New York, New York; 5 Memorial Sloan Kettering Cancer Center, New York, New York

## Abstract

**Importance:**

Initial management of intermediate-risk prostate cancer is evolving, with no clear recommendation for treatment. Data on utilization of active surveillance for patients with newly diagnosed intermediate-risk prostate cancer may help clarify emerging trends.

**Objective:**

To further characterize US national trends of initial management of intermediate-risk prostate cancer.

**Design, Setting, and Participants:**

This cohort study included patients with intermediate-risk prostate cancer diagnosed from January 1, 2010, to December 31, 2020. Eligible patients were diagnosed in US hospitals included in the National Cancer Database; National Comprehensive Cancer Network risk stratification guidelines were used to characterize as favorable vs unfavorable intermediate risk. Analysis was performed in September 2023.

**Exposure:**

Active surveillance vs intervention with surgery and/or radiation or no treatment.

**Main Outcomes and Measures:**

Temporal trends in demographic, clinical, and socioeconomic factors among men with intermediate-risk prostate cancer and their association with the use of active surveillance; further subgroup analysis was conducted for those with favorable vs unfavorable intermediate risk classification.

**Results:**

In total, 289 584 men diagnosed with intermediate-risk prostate cancer were identified from 2010 to 2020 (46 147 Black [15.9%], 230 071 White [79.5%]). Among patients, 153 726 (53.1%) underwent prostatectomy, 107 152 (37.0%) underwent radiotherapy, and 15 847 (5.5%) underwent active surveillance as initial treatment strategy. Overall, active surveillance quadrupled from 418 of 21 457 patients (2.0%) in 2010 to 2428 of 28 192 patients (8.6%) in 2020 for the entire cohort (*P* < .001). Active surveillance increased from 317 of 12 858 patients (2.4%) in 2010 to 2020 of 12 902 patients (13.5%) in 2020 in men with favorable intermediate-risk prostate cancer (*P* < .001). In the unfavorable intermediate-risk cohort, active surveillance increased from 101 of 8181 patients (1.2%) in 2010 to 408 of 12 861 patients (3.1%) in 2020 (*P* < .001). On multivariable analysis, use of active surveillance was associated with increased age (age 70-80 years vs <50 years: odds ratio [OR], 3.09; 95% CI, 2.66-3.59), lower Gleason score (3 + 3 vs 3 + 4: OR, 3.45; 95% CI, 3.25-3.66), early T stage (T2c vs T1a through T2a: OR, 0.35; 95% CI, 0.32-0.38), treatment at an academic center (community vs academic center: OR, 0.72; 95% CI, 0.67-0.78), higher level of education (communities with 21% or higher population without high school vs less than 7%: OR, 0.73; 95% CI, 0.67-0.79), insurance type (Medicare or other governmental service vs private: OR, 1.11; 95% CI, 1.07-1.16), proximity to treatment facility (greater than 120 miles vs less than 60 miles: OR, 0.75; 95% CI, 0.68-0.84), facility location (South Atlantic vs New England: OR, 0.54; 95% CI, 0.46-0.53), and lower income (less than $38 000 vs $63 000 or greater: OR, 1.22; 95% CI, 1.14-1.31).

**Conclusions and Relevance:**

These findings highlight increasing implementation of active surveillance in the initial management of intermediate risk prostate cancer. Prospective data with improved risk stratification incorporating genomics and digital pathology artificial intelligence as well as novel surveillance strategies may continue to better delineate optimal treatment recommendations in this patient population.

## Key Points

**Question:**

What clinical factors and sociodemographic patient characteristics are associated with utilization of active surveillance for the primary management of intermediate-risk prostate cancer?

**Findings:**

In this cohort study using data from 289 584 patients identified in the National Cancer Database, the utilization of active surveillance as initial management strategy for patients newly diagnosed with intermediate-risk prostate cancer has increased over time. On multivariable analysis, the use of active surveillance was associated with increased age, lower Gleason score, early T stage, treatment at an academic center, higher level of education, government provided insurance type, closer proximity to treatment facility, regional facility location, and lower income.

**Meaning:**

Our findings suggest active surveillance utilization is increasing over time with key clinical and sociodemographic factors associated with this management strategy.

## Teaser

This cohort study examines trends in the demographic and clinical characteristics of US men newly diagnosed with intermediate-risk prostate cancer whose treatment utilized active surveillance.

## Introduction

In 2023, prostate cancer remains a significant health burden, with an estimated 288 300 new cases and 34 700 deaths in the US.^1^ For men with a new diagnosis, optimal treatment management depends on a variety of factors related to patients’ age, prostate-specific antigen (PSA) levels, tumor stage, and pathology from standardized multicore biopsy. Risk stratification systems, including those published in Nation Comprehensive Cancer Network (NCCN) guidelines, stratify patients based on these patient oncologic characteristics, with management options tailored to a patient’s estimated life expectancy.^2^ Current NCCN recommendations allow for active surveillance or definitive management with surgery or radiation for patients with favorable intermediate risk prostate cancer, with similar recommendations—sans active surveillance and with or without androgen deprivation therapy (ADT) intensification—for those with unfavorable risk disease. Data from several prospective trials including PIVOT and UK ProtecT demonstrate comparably low prostate cancer–specific mortality for patients with low risk disease, or those diagnosed by PSA screening, randomized to active treatment with prostatectomy, radiation therapy, or observation at initial prostate cancer diagnosis.^3,4^ Active surveillance is the preferred strategy for men with low-risk prostate cancer, and use of this strategy has increased over time.^5^ With the inclusion of an additional 4 years of data, this study is an update of our prior research reexamining national trends in implementation of active surveillance as an initial treatment strategy in men with intermediate-risk prostate cancer.^6^

## Methods

### Data Source

The National Cancer Database (NCDB) is a joint project of the Commission on Cancer (CoC) of the American College of Surgeons and the American Cancer Society. The NCDB tabulates longitudinal data from more than 70% of all new cancer diagnoses on an annual basis, encompassing more than 1500 hospitals across all 50 states. From 2010 to 2020, men undergoing active surveillance were reported to the NCDB and coded within the variable *RX_SUMM_TREATMENT_STATUS*. The collected data include cancer characteristics, primary and adjuvant management, and long-term outcomes, as well as self-reported patient demographic information such as age, sex, race, educational level, income, insurance status, Charlson-Deyo comorbidity score, facility type, distance from facility, and facility geographic region. As this study used deidentified data from the NCDB, the requirement for formal institutional review and the need for informed patient consent were waived, consistent with the policies of Weill Cornell Medicine. The CoC’s NCDB and the hospitals participating in the NCDB are the source of the deidentified data used herein; they have not verified and are not responsible for the statistical validity of the data analysis or the conclusions derived by the authors. This report follows the Strengthening the Reporting of Observational Studies in Epidemiology (STROBE) reporting guideline for cohort studies.

### Study Population

Men with intermediate-risk prostate cancer coded for active surveillance, surgery, or radiotherapy as the initial treatment strategy from 2010 to 2020 were included in this study (eFigure in Supplement 1). All risk groups referenced in this study are based on NCCN guidelines accessed during analysis.^2^ Men classified as low risk or high risk for prostate cancer were excluded from analysis. Intermediate risk factors include Gleason score of 7 (3 + 4 or 4 + 3), PSA 10 to 20 ng/mL, or clinical stage cT2b or cT2c. Further stratification into favorable intermediate risk and unfavorable intermediate risk was performed for subgroup analysis. Favorable intermediate risk was defined as the presence of only 1 intermediate risk factor and Gleason score other than 4 + 3. Unfavorable intermediate risk was defined as the presence of a Gleason score of 4 + 3 or the presence of at least 2 intermediate risk factors.

### Active Surveillance Patient Selection

Only men coded for active surveillance (ie, *RX_SUMM_TREATMENT_STATUS*) were considered to have undergone active surveillance. Men who did not receive any primary therapy (radiation or surgery) were not considered to have undergone active surveillance. Duration of active surveillance or details of any subsequent treatment were not available for analysis.

### Statistical Analysis

Descriptive statistics for factors of interest were reported as frequency. The Cochran-Armitage test was used to identify significant trends in active surveillance or intervention (prostatectomy or radiotherapy). Multivariable logistic regression was performed to examine demographic, clinical, and socioeconomic factors associated with active surveillance or intervention. The logistic regression was adequate to discriminate whether the patient got active surveillance (area under the curve = 0.815). Analysis was performed on the entire cohort as well as for favorable and unfavorable intermediate risk subgroups. All tests were 2-sided and considered significant at a level of *P* < .05. All analyses were performed using SAS software, version 9.4 (SAS Institute). Analysis was performed in September 2023.

## Results

In total, 289 584 men were identified with intermediate-risk prostate cancer from 2010 to 2020 (mean [SD] age, 64.5 [7.7] years; 46 147 Black [15.9%], 230 071 White [79.5%]) (Table 1). A total of 236 895 men (81.8%) in the cohort had a Charlson-Deyo comorbidity index score of 0. Of these men, 153 726 (53.1%) underwent prostatectomy, 107 152 (37.0%) underwent radiotherapy, and 15 847 (5.5%) underwent active surveillance. Overall, active surveillance quadrupled from 418 out of 21 457 patients (2.0%) in 2010 to 2428 out of 28 191 (8.6%) in 2020 (*P* < .001). Active surveillance increased from 317 out of 12 858 patients (2.4%) in 2010 to 2020 out of 12902 patients (13.5%) in 2020 in men with favorable intermediate-risk prostate cancer (*P* < .001) (Figure). In the unfavorable intermediate-risk cohort, active surveillance increased from 101 out of 8181 patients (1.2%) in 2010 to 408 out of 12 861 patients (3.1%) in 2020 (*P* < .001). The mean (SD) age in the active surveillance and treatment cohorts was 67.8 (8.1) years and 64.3 (7.6) years, respectively.

**Table 1.** Patient Demographics

Characteristics	Patients, No. (%)			*P* value
	Overall (n = 289 584)	Intervention (n = 273 737)	Active surveillance (n = 15 847)	
Treatment				
Active surveillance	15 847 (5.5)	15 847 (5.5)	15 847 (100.0)	NA
Surgery	153 726 (53.1)	153 726 (56.2)	0	
Radiotherapy	107 152 (37.0)	107 152 (39.1)	0	
Other	12 859 (4.4)	12 859 (4.7)	0	
Year of diagnosis				
2010	21 457 (7.4)	21 039 (7.7)	418 (2.6)	<.001
2011	23 347 (8.1)	22 741 (8.3)	606 (3.8)	
2012	20 724 (7.2)	20 140 (7.4)	584 (3.7)	
2013	21 179 (7.3)	20 298 (7.4)	881 (5.6)	
2014	20 795 (7.2)	19 968 (7.3)	827 (5.2)	
2015	24 217 (8.4)	23 070 (8.4)	1147 (7.2)	
2016	28 020 (9.7)	26 464 (9.7)	1556 (9.8)	
2017	31 725 (11.0)	29 666 (10.8)	2059 (13.0)	
2018	33 038 (11.4)	30 620 (11.2)	2418 (15.3)	
2019	36 891 (12.7)	33 968 (12.4)	2923 (18.5)	
2020	28 191 (9.7)	25 763 (9.4)	2428 (15.3)	
Age, mean (SD), y	64.5 (7.7)	64.3 (7.6)	67.8 (8.1)	<.001
Age				
<50 y	7129 (2.5)	6921 (2.5)	208 (1.31)	<.001
50-60 y	68 532 (23.7)	66 228 (24.2)	2304 (14.5)	
60-70 y	137 896 (47.6)	131 189 (47.9)	6707 (42.3)	
70-80 y	69 910 (24.1)	64 421 (23.5)	5489 (34.6)	
80-90 y	6117 (2.1)	4978 (1.8)	1139 (7.2)	
Gleason score				
3 + 3	27 989 (9.7)	21 602 (7.9)	6387 (40.3)	<.001
3 + 4	177 204 (61.2)	169 104 (61.8)	8100 (51.1)	
4 + 3	84 391 (29.1)	83 031 (30.3)	1360 (8.6)	
PSA, median (IQR)	6.5 (4.9-9.5)	6.4 (4.9-9.3)	8.9 (5.6-11.8)	<.001
PSA				
<4	31 192 (10.8)	30 063 (11.0)	1129 (7.1)	<.001
4-10	194 239 (67.1)	186 456 (68.1)	7783 (49.1)	
10-20	64 153 (22.15)	57 218 (20.9)	69,35 (43.8)	
Clinical T stage				
T1a-T2a	254 508 (87.9)	239 864 (87.6)	14 644 (92.4)	<.001
T2b	14 631 (5.1)	14 166 (5.2)	465 (2.9)	
T2c	20 445 (7.1)	19 707 (7.2)	738 (4.7)	
Charlson-Deyo comorbidity index score				
0	236 895 (81.8)	223 673 (81.7)	13 222 (83.4)	<.001
1	39 965 (13.8)	38 202 (14.0)	1763 (11.1)	
2	8324 (2.9)	7807 (2.9)	517 (3.3)	
≥3	4400 (1.5)	4055 (1.5)	345 (2.2)	
Race				
Black	46 147 (15.9)	43 719 (16.0)	2428 (15.3)	<.001
White	23 0071 (79.5)	21 7576 (79.5)	12 495 (78.9)	
Other^a^	13 366 (4.6)	12 442 (4.6)	924 (5.8)	
Primary payer				
Medicare/Medicaid/other government	146 390 (50.6)	136 725 (50.0)	9665 (61.0)	<.001
Private	136 629 (47.2)	130 890 (47.8)	5739 (36.2)	
Uninsured	3473 (1.2)	3203 (1.2)	270 (1.7)	
Unknown	3092 (1.1)	2919 (1.1)	173 (1.1)	
Facility location				
New England	18 787 (6.5)	17 236 (6.3)	1551 (9.8)	<.001
Mid Atlantic	46 165 (15.9)	43 499 (15.9)	2666 (16.8)	
South Atlantic	62 543 (21.6)	59 855 (21.9)	2688 (17.0)	
Central (East North)	50 409 (17.4)	47 378 (17.3)	3031 (19.1)	
Central (East South)	19 511 (6.7)	18 948 (6.9)	563 (3.6)	
Central (West North)	25 308 (8.7)	23 971 (8.8)	1337 (8.4)	
Central (West South)	19 227 (6.6)	18 272 (6.7)	955 (6.0)	
Mountain	11 442 (4.0)	10 796 (3.9)	646 (4.1)	
Pacific	36 192 (12.5)	33 782 (12.3)	2410 (15.2)	
Facility type				
Community	16 134 (5.6)	15 061 (5.5)	1073 (6.8)	<.001
Comprehensive	108 011 (37.3)	103 396 (37.8)	4615 (29.1)	
Academic	113 278 (39.1)	105 529 (38.6)	7749 (48.9)	
Integrated	52 161 (18.0)	49 751 (18.2)	2410 (15.2)	
Median annual income				
<$38 000	42 184 (14.6)	39 994 (14.6)	2190 (13.8)	.008
$38 000-$47 999	59 083 (20.4)	55 839 (20.4)	3244 (20.5)	
$48 000-$62 999	68 578 (23.7)	64 883 (23.7)	3695 (23.3)	
≥$63 000	119 739 (41.4)	113 021 (41.3)	6718 (42.4)	
Education level (no high school)				
≥21%	49 576 (17.1)	47 042 (17.2)	2534 (16.0)	<.001
13%-20.9%	75 719 (26.2)	71 731 (26.0)	3988 (25.2)	
7%-12.9%	87 822 (30.3)	83 024 (30.3)	4798 (30.3)	
<7% (highest)	76 467 (26.4)	71 940 (26.3)	4527 (28.6)	
Distance from facility				
<60 mi	258 707 (89.3)	244 297 (89.3)	14 410 (90.9)	<.001
60-120 mi	19 044 (6.6)	18 132 (6.6)	912 (5.8)	
≥120 mi	11 833 (4.1)	11 308 (4.1)	525 (3.3)	
Location type				
Metropolitan	244 093 (84.3)	230 686 (84.3)	13 407 (84.6)	.44
Urban	40 098 (13.9)	37 937 (13.9)	2161 (13.6)	
Rural	5393 (1.9)	5114 (1.9)	279 (1.76)	
Intermediate-risk group				
Favorable	163 480 (56.5)	15 053 (55.0)	12 944 (81.7)	<.001
Unfavorable	126 104 (43.6)	123 201 (45.0)	2903 (18.3)	

Abbreviation: PSA, prostate-specific antigen.

^a^ Other includes American Indian, Aleutian or Eskimo, Chinese, Japanese, Filipino, Hawaiian, Korean, Vietnamese, Laotian, Hmong, Kampuchean, Thai, Asian Indian or Pakistani, Asian Indian, Pakistani, Micronesian, Chamorro, Guamanian, Polynesian, Tahitian, Samoan, Tongan, Melanesian, Fiji Islander, New Guinean, other Asian (including Asian, not otherwise specified and Oriental, not otherwise specified and Pacific Islander, not otherwise specified, other, and unknown).

**Figure.**
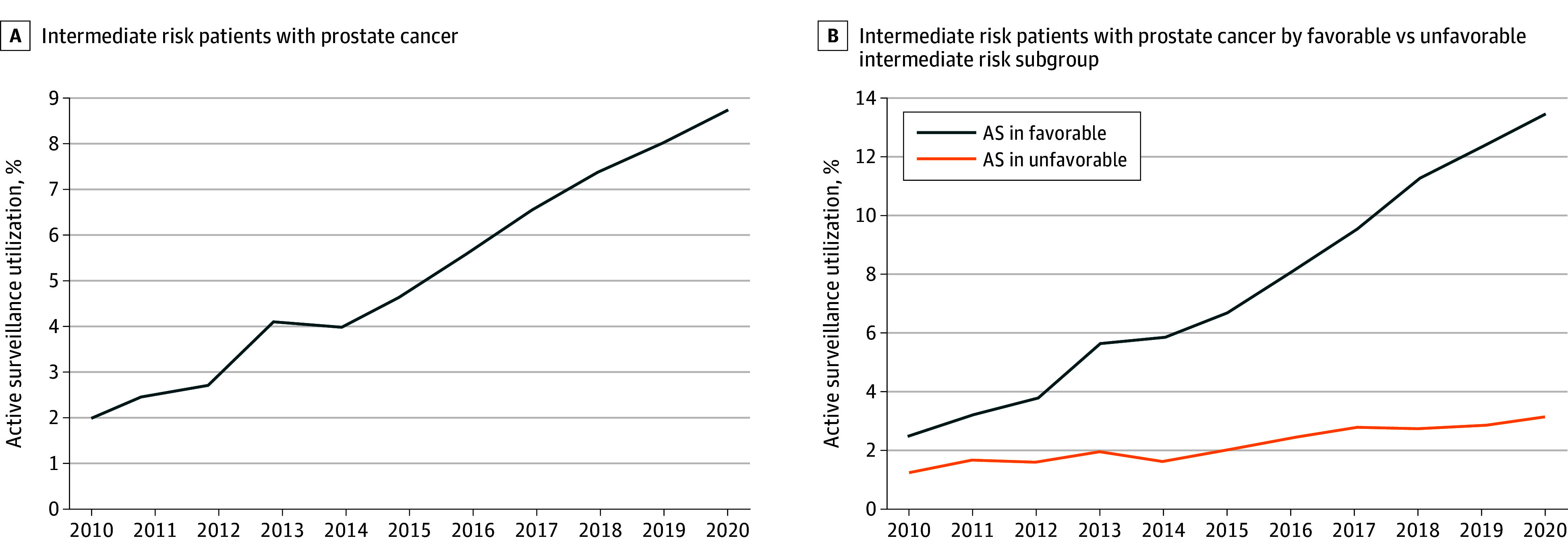
Trends in Active Surveillance (AS) Utilization as Initial Treatment Strategy for Patients With Intermediate-Risk Prostate Cancer

In multivariable analyses for men with intermediate-risk prostate cancer undergoing active surveillance, older men were more likely to undergo active surveillance compared with men aged 50 years or younger (the reference) (aged 50-60 years: OR, 1.13 [95% CI, 0.98-1.32]; aged 60-70 years: OR, 1.69 [95% CI, 1.46-1.96]; aged 70-80 years: OR, 3.09 [95% CI, 2.66-3.59]; aged 80-90 years: OR, 11.12 [95% CI, 9.42-13.12]) (Table 2). Men diagnosed after 2010 (the reference) were more likely to undergo active surveillance (2020: OR, 7.20 [95% CI, 6.45-8.05]). Compared with men with Gleason scores of 4 + 3, men with lower scores were more likely to undergo active surveillance (Gleason score 3 + 3: OR, 36.44 [95% CI, 33.88-39.20]; Gleason score 3 + 4: OR, 3.45 [95% CI, 3.25-3.66]). Men who presented with clinically higher tumor stage were less likely to undergo active surveillance (cT2b: OR, 0.35 [95% CI, 0.32-0.39]; cT2c: OR, 0.35 [95% CI, 0.32-0.38]).

**Table 2.** Multivariable Analysis

Parameter	OR (95% CI)	*P* value
Year of diagnosis		
2010	1 [Reference]	<.001
2011	1.40 (1.23-1.60)	
2012	1.76 (1.55-2.01)	
2013	2.71 (2.39-3.06)	
2014	2.60 (2.30-2.95)	
2015	3.25 (2.88-3.66)	
2016	4.08 (3.64-4.58)	
2017	4.97 (4.44-5.55)	
2018	5.62 (5.03-6.28)	
2019	6.33 (5.68-7.06)	
2020	7.20 (6.45-8.05)	
Age		
<50 y	1 [Reference]	<.001
50-60 y	1.134 (0.98-1.32)	
60-70 y	1.69 (1.46-1.96)	
70-80 y	3.09 (2.66-3.59)	
80-90 y	11.12 (9.42-13.12)	
Gleason score		
4 + 3	1 [Reference]	<.001
3 + 4	3.45 (3.25-3.66)	
3 + 3	36.44 (33.88-39.2)	
Clinical T stage:		
T1a-T2a	1 [Reference]	<.001
T2b	0.35 (0.32-0.39)	
T2c	0.35 (0.32-0.38)	
Race		
White	1 [Reference]	<.001
Black	1.10 (1.04-1.16)	
Other^a^	1.15 (1.06-1.24)	
Primary payer		
Private	1 [Reference]	<.001
Medicare, Medicaid, or other government	1.11 (1.07-1.16)	
Uninsured	1.67 (1.45-1.92)	
Unknown	1.10 (0.93-1.30)	
Facility location		
New England	1 [Reference]	<.001
Central (East North)	0.64 (0.60-0.69)	
Central (East South)	0.32 (0.29-0.36)	
Mid Atlantic	0.60 (0.56-0.65)	
Mountain	0.72 (0.65-0.8)	
Pacific	0.82 (0.77-0.89)	
South Atlantic	0.49 (0.46-0.53)	
Central (West North)	0.63 (0.58-0.68)	
Central (West South)	0.54 (0.49-0.59)	
Facility type		
Academic	1 [Reference]	<.001
Community	0.72 (0.67-0.78)	
Comprehensive	0.52 (0.50-0.55)	
Integrated	0.61 (0.58-0.65)	
Median annual income		
≥$63 000	1 [Reference]	<.001
$48 000-$62 999	1.09 (1.04-1.15)	
$38 000-$47 999	1.22 (1.15-1.29)	
<$38 000	1.22 (1.14-1.31)	
Education level, no high school		
<7% (highest)	1 [Reference]	<.001
7%-12.9%	0.92 (0.87-0.97)	
13%-20.9%	0.83 (0.77-0.88)	
≥21%	0.73 (0.67-0.79)	
Distance from facility		
<60 mi	1 [Reference]	<.001
60-120 mi	0.82 (0.75-0.89)	
≥120 mi	0.75 (0.68-0.84)	

Abbreviation: OR, odds ratio.

^a^ Other includes American Indian, Aleutian or Eskimo, Chinese, Japanese, Filipino, Hawaiian, Korean, Vietnamese, Laotian, Hmong, Kampuchean, Thai, Asian Indian or Pakistani, Asian Indian, Pakistani, Micronesian, Chamorro, Guamanian, Polynesian, Tahitian, Samoan, Tongan, Melanesian, Fiji Islander, New Guinean, and other Asian (including Asian, not otherwise specified and Oriental, not otherwise specified and Pacific Islander, not otherwise specified, other, and unknown).

Men with no insurance or government insurance were more likely to undergo active surveillance than private insurance (government: OR, 1.11 [95% CI, 1.07-1.16]; uninsured: OR, 1.67 [95% CI, 1.45-1.92]). Men treated outside of an academic program were less likely to undergo active surveillance (comprehensive program: OR, 0.52 [95% CI, 0.50-0.55]; integrated program: OR, 0.61 [95% CI, 0.58-0.65]; community program: OR, 0.72 [95% CI, 0.67-0.78]). Patients who live in an area outside of New England were less likely to undergo active surveillance (East North Central: OR, 0.64 [95% CI, 0.60-0.69]; East South Central: OR, 0.32 [95% CI, 0.29-0.36]; Middle Atlantic: OR, 0.60 [95% CI, 0.56-0.65]; Mountain: OR, 0.72 [95% CI, 0.65-0.80]; Pacific: OR, 0.82 [95% CI, 0.77-0.89]; South Atlantic: OR, 0.49 [95% CI, 0.46-0.53]; West North Central: OR, 0.63 [95% CI, 0.58-0.68]; West South Central: OR, 0.54 [95% CI, 0.49-0.59]). Men who lived in a rural area were more likely to undergo active surveillance than those living in a metropolitan area (OR, 1.18 [95% CI, 1.03-1.36]). Men who lived farther from a treatment facility were less likely to undergo active surveillance (60 to 120 miles: OR, 0.81 [95% CI, 0.75-0.87]; greater than 120 miles: OR, 0.75 [95% CI, 0.68-0.83]). Men in regions with increased high school completion were less likely to undergo active surveillance (communities with 21% or higher population without high school completion vs less than 7%: OR, 0.73 [95% CI, 0.67-0.79]).

## Discussion

Using a national hospital-based, oncology-focused database cohort of patients newly diagnosed with prostate cancer, we show active surveillance utilization is increasing over time in those with intermediate risk disease, consistent with our prior analysis.^6^ Our results suggest that approximately 8.6% of patients with intermediate-risk prostate cancer elected for active surveillance as their primary management strategy in 2020, with a preponderance of those with favorable intermediate risk comprising this group. This rate has increased significantly compared with 2.0% of men in 2010, but remains lower than rates recorded in other countries. According to the National Prostate Cancer Register of Sweden (NPCR), in 2022, 17% of intermediate-risk cases underwent active surveillance.

Our updated analysis highlights several predictable and previously reported cancer and patient specific factors associated with patients undergoing active surveillance. These factors include increased patient age, grade group 1 or 2 pathology, and earlier T stage. Our data highlights that most patients undergoing active surveillance have a low Charlson-Deyo comorbidity score and that the decision regarding definitive treatment or active surveillance is not driven by poor performance status. Our analysis also highlights independent socioeconomic and demographic factors such as government insurance or lack of insurance, treatment at academic institutions, treatment at facilities located in New England, lower income, non-White race, patient proximity to treatment location, and patient residence in rural areas or areas with lower high school completion percentage as independent factors that affect the odds of patients receiving active surveillance. How these demographic and socioeconomic factors may affect active surveillance implementation, patient selection, and treatment discussions between clinicians and patients is worth consideration, although out of scope of this article.

The 2023 15-year follow-up publication from the UK ProtecT study shows comparable, noninferior prostate cancer–specific mortality between patients randomized to prostatectomy, definitive radiation or observation suggesting the safety and practicality of active surveillance for patients with newly diagnosed, organ-confined prostate cancer.^4^ Even though 61% of men originally assigned to active surveillance had moved to radical treatment and the fact that there were higher rates of metastasis in the observation arm, these factors did not portend a statistically worse overall outcome between the arms examined in the study. Furthermore, there are a portion of patients randomized to the observation arm who are still alive and have yet to receive any treatment for their cancer and have thus far avoided morbidity associated with definitive treatment. However, several of the patients randomized to the observation arm died from their cancer, necessitating caution with blanket recommendations for surveillance for all comers.

Additional data points beyond PSA, Gleason score or pathology, physical examination, and age might be necessary to better characterize and predict which patients would benefit from definitive treatment and what level of treatment intensification or deintensification may lead to superior outcomes. How underlying genomic risk may play a role is yet to be solidified for patients considering active surveillance, although studies suggest implementation of available genomic tests may better risk stratify patients and inform decision making.^7^ There are 2 large randomized NRG clinical trials (GU-010 and GU-009) implementing genomic risk for treatment intensification or deintensification in patients with unfavorable intermediate-risk and high-risk prostate cancer undergoing definitive radiation therapy, respectively. Whether there will be similar strategies for those considering active surveillance is intriguing. In addition to genomic tests, there are ongoing efforts utilizing artificial intelligence models that are promising and reportedly statistically superior at stratifying patients risk and cancer related outcomes when compared with D’Amico risk groups utilized in current NCCN guidelines.^8^

Advances in imaging including the integral role of multiparametric magnetic resonance imaging (MRI) has allowed for more accurate targeting of potentially significant prostate cancer and monitoring of patients on active surveillance.^9^ There are recent studies suggesting prostate-specific membrane antigen PET/CT combined with mpMRI allows for improved negative predictive value and sensitivity for clinically significant prostate cancer in an MRI-triaged population thus potentially reducing need for prostate biopsies for some patients.^10^ Whether prostate-specific membrane antigen PET/CT can be implemented for monitoring patients on active surveillance is of interest, particularly for those patients unable to undergo MRI or those without significant changes on imaging. How best to incorporate these new developments across a broad spectrum of patients remains to be seen but will certainly offer more information for the clinician to guide recommendations for patients presenting with intermediate-risk prostate cancer exploring all available options.

### Limitations

Limitations of this study include those associated with its retrospective nature and analysis of a dataset that includes information from a national hospital-based registry. As such, this is not a population-based cohort and there is a possibility that the numbers reported based on this dataset do not accurately or precisely reflect the entirety of patients with newly diagnosed prostate cancer in the US. Furthermore, the database collects only the initial treatment course of the patients and does not account for subsequent treatment decisions or information. For instance, a patient recommended to undergo active surveillance and coded as such may ultimately proceed with alternative treatment at their discretion, which would not be included or represented in the data available for analysis.

## Conclusions

In this cohort study of patients with intermediate-risk prostate cancer diagnosed from 2010 to 2020, we provided an updated analysis highlighting the increasing implementation of active surveillance as an initial treatment approach. Furthermore, our subset analysis shows this is particularly evident for those considered to be favorable intermediate risk. Prospective data with improved risk stratification incorporating genomics as well as novel surveillance strategies may continue to better delineate ideal or optimal candidates for this treatment approach thereby doing less harm while continuing to treat a deadly disease.

## Notes

Supplement 1. **eFigure.** Study Flow Diagram

Supplement 2.
Data Sharing Statement
