# Supplementary material for: Trends in Active Surveillance for Men With Intermediate-Risk Prostate Cancer
Source: JAMA Netw Open. 2024 Aug 22;7(8):e2429760. doi: 10.1001/jamanetworkopen.2024.29760 (PMC11342134; doi:10.1001/jamanetworkopen.2024.29760)
Supplement: Supplement 1. — eFigure. Study Flow Diagram [file jamanetwopen-e2429760-s001.pdf]

Supplemental Online Content

Diven MA, Tshering L, Ma X, et al. Trends in active surveillance for men with intermediate risk prostate cancer. *JAMA Netw Open.* 2024;7(8):e2429760. doi:10.1001/jamanetworkopen.2024.29760

eFigure. Study Flow Diagram

This supplemental material has been provided by the authors to give readers additional information about their work.

© 2024 Diven MA et al. *JAMA Network Open.*

Supplemental Figure 1. Study Flow Diagram

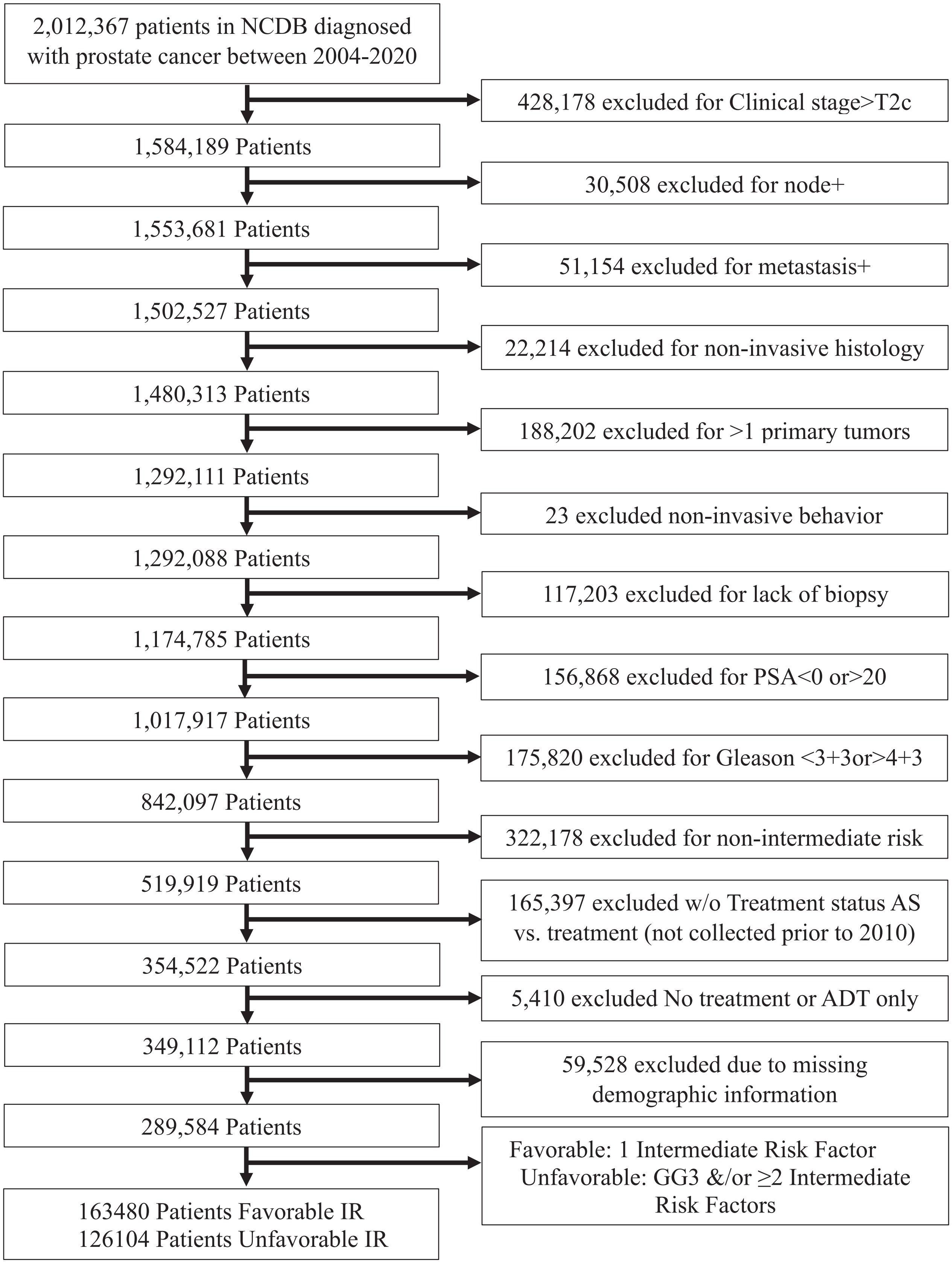

© 2024 Diven MA et al. *JAMA Network Open.*
