# Supplementary material for: Trends in Active Surveillance for Men With Intermediate-Risk Prostate Cancer
Source: JAMA Netw Open. 2024 Aug 22;7(8):e2429760. doi: 10.1001/jamanetworkopen.2024.29760 (PMC11342134; doi:10.1001/jamanetworkopen.2024.29760)
Supplement: Supplement 2. — Data Sharing Statement [file jamanetwopen-e2429760-s002.pdf]

Data Sharing Statement

Data
Data available: No

Additional Information
Explanation for why data not available: NCDB data base is controlled by ACS and is available upon request and application submission. Methods for our analysis would be available at request.
